# Phenotypes and Genotypes of Patients with Pantothenate Kinase-Associated Neurodegeneration in Asian and Caucasian Populations: 2 Cases and Literature Review

**DOI:** 10.1155/2013/860539

**Published:** 2013-11-19

**Authors:** Chih-Hong Lee, Chin-Song Lu, Wen-Li Chuang, Tu-Hsueh Yeh, Shih-Ming Jung, Chia-Ling Huang, Szu-Chia Lai

**Affiliations:** ^1^Department of Neurology, Chang Gung Memorial Hospital at Linkou Medical Center and Chang Gung University, Taoyuan 333, Taiwan; ^2^Neuroscience Research Center, Chang Gung Memorial Hospital at Linkou Medical Center, Taoyuan 333, Taiwan; ^3^Department of Pathology, Chang Gung Memorial Hospital at Linkou Medical Center and Chang Gung University, Taoyuan 333, Taiwan; ^4^Department of Neurology, Saint Paul's Hospital, Taoyuan 333, Taiwan

## Abstract

*Objectives*. Pantothenate kinase-associated neurodegeneration (PKAN) is a rare disease caused by pantothenate kinase 2 (*PANK2*, OMIM 606157) mutations. This study is aimed to investigate clinical presentations, pathologies, and genetics in patients with PKAN. *Methods*. Two patients with PKAN were reported. We reviewed the literature to include additional 19 patients with PKAN in Eastern Asia. These patients were divided into classic and atypical groups by the age of onset. We compared the data on PKAN patients of Asian and Caucasian populations. *Results*. We found iron deposits in the globus pallidus in our Patient 1 and a heterozygous truncating mutation (c.1408insT) in Patient 2. Literature review shows that generalized dystonia and bulbar signs are more common in classic PKAN patients, whereas segmental dystonia and tremors are more specific to atypical ones. Asian patients have less complex presentations—lower prevalence of pyramidal signs, mental impairment, and parkinsonism—than Caucasians. D378G in exon 3 is the most frequent mutation (28%) in Asians. *Conclusions*. Our study demonstrates that the distribution of dystonia is the major distinction between subgroups of PKAN. Caucasian patients have more complex presentations than Asians. Exon 3 and 4 are hot spots for screening *PANK2* mutations in Asian patients.

## 1. Introduction

 Neurodegeneration with brain iron accumulation (NBIA) is a syndrome comprising heterogeneous groups of diseases characterized by iron deposition in the basal ganglia. This group has different clinical presentations and genetics but shares the same characteristics of iron accumulation in the brain. In recent years, several genetic causes have been identified, such as *PANK2*, *PLA2G6*, *FA2H*, *ATP13A2*, *CP*, and *FTL* [1]. Among them, the core syndromes are pantothenate kinase-associated neurodegeneration (PKAN, NBIA type 1), which was previously named Hallervorden-Spatz disease and accounts for approximately 50% of NBIA cases [2], and *PLA2G6*-associated neurodegeneration (PLAN, NBIA type 2). 

 PKAN is classified as classic or atypical according to the age of onset, rate of progression and severity of the motor symptoms [3]. Classic presentations include dystonia, gait disturbance, pyramidal and extrapyramidal involvement with early onset (in the first decade), and rapid progression. In atypical PKAN, the age of onset is relatively later (in the second decade or later), and the progression is slower. Motor and gait abnormalities are less severe. Patients may still ambulate decades after the disease onset. In 2001, the causative gene of PKAN, *PANK2*, was first identified to be located on chromosome 20p13 [4]. Since then, more than 100 mutations have been published. *PANK2* encodes a 1.85 kb transcript that includes 7 exons. A genotype and phenotype correlation study showed that all patients with classic PKAN and one-third of the atypical group had *PANK2* mutations [3]. Two mutations in the *PANK2* gene, G521R and T528M, have been found to be common in people of European descent, found in one-third of the patients with PKAN [3]. 

 Ethnic differences play an important role in human diseases, both clinically and genetically. We herein present the clinical features, genetic analysis, electrophysiological studies, and neuroimaging of 2 Taiwanese patients with PKAN. We also compare the data on PKAN patients of Asian and Western populations from the literature, focusing on clinically and genetically distinguishing features in classic and atypical cases.

## 2. Patients and Methods

### 2.1. Case Reports

#### 2.1.1. Patient 1

This Taiwanese man developed tremors in both hands at the age of 23 years. The tremors slowly spread to his legs, head, and mouth, followed by slurred speech and gait disturbance. None of his family members had similar symptoms. Ten years after the disease onset, large-amplitude, asymmetric jerky action tremors that were more significant in the right proximal upper extremity were observed, along with generalized slowness and rigidity, tongue dyskinesia, dysarthria, and an unsteady gait. He did not respond to levodopa. He became wheelchair bound 25 years after the onset of symptoms. His cognitive function was relatively preserved until the final years before his death. He died due to aspiration pneumonia at the age of 56 years. He received postmortem autopsy.

#### 2.1.2. Patient 2

 This 36-year-old Taiwanese man developed intermittent tremors in his right arm at the age of 18 years. The tremors occurred with activity and could be exacerbated by anxiety. The tremor worsened and interfered with his daily activities such as eating when he was 35 years old. In addition, torsion of the right upper limb was noticed while writing or walking activities. He also began stuttering, but he did not have difficulty in swallowing, limb weakness, or gait disturbance. 

 Neither patient showed a Kayser-Fleischer ring or retinal pigmentation. Serum levels of ceruloplasmin, copper, and creatine kinase and liver function test were within normal limits. Brain magnetic resonance images (MRI) from both patients showed the characteristic “eye-of-the-tiger” sign.

### 2.2. Genetic Analysis

Genomic DNA was prepared from white blood cells from the peripheral blood of all suspected patients and controls using the Puregene DNA purification kit from GENTRA (Minneapolis, MN). Polymerase chain reaction (PCR) amplification was performed using an Applied Biosystems 9600 Thermal Cycler. PCR products were purified using the Concert Rapid PCR Purification System (Invitrogen, Carlsbad, CA). Mutations were verified by automated sequencing using dye terminator chemistry in the Automated Sequencer Genetic Analyzer model 3100 (Applied Biosystems, CA, USA). The Autoassembler computer program (Applied Biosystems) was used for sequence alignments and analysis. All the primer sequences used in this study are available upon request.

To assess for exonic deletion or duplication, multiple-ligation probe amplification (MLPA) analysis was performed with 100 ng of genomic DNA, according to the manufacturer's instructions. The SALSA MLPA kit P120 *PANK2*/*PLA2G6* test kit (MRC-Holland; Amsterdam, The Netherlands) contains 1 probe for each exon of *PANK2* and 2 probes for exons 1, 2, 4, and 6. No probes are available for exon 3, which is located very close to exon 2 and is only present in the transcript variant 3. Probe amplification products were run on an ABI 3730 XL DNA Analyzer by using the GS500 size standard (Applied Biosystems). MLPA peak plots were visualized using GeneMapper Software version 3.7 (Applied Biosystems). Relative peak area values of the affected members were compared to those of healthy controls and were expressed as percent ratios and bar charts (Microsoft Excel). 

Informed consent was obtained from all participants, and the study was approved by the Institutional Review Board of Chang Gung Memorial Hospital (Approval no. 100-3931A3).

### 2.3. Literature Review

To know the clinical and genetic differences of PKAN patients between Asians and Caucasians, we reviewed the literature for including PKAN patients in Eastern Asia in our study. We used the diagnostic criteria proposed by Swaiman [5, 6] as the inclusion criteria for this study, except for the age at onset because some of the patients had adult-onset diseases. Essential features required for the diagnosis of NBIA were progressive disorder associated with at least one of the following conditions: dystonia, rigidity, tremor, bradykinesia, or choreoathetosis, as well as the presence of at least one of the following supporting evidence items: the “eye-of-the-tiger” sign by MRI (hyperintensity at the globus pallidus on T1-weighted images and hypointensity with central hyperintensity on T2-weighted images), genetic proof of *PANK2* mutations, or pathology-proved autopsies. We summarized and analyzed the clinical and laboratory information from the reports. Patients without a detailed history were excluded, and the remaining patients were divided into classic (onset before 10 years) and atypical (onset after 10 years of age) groups. We also compared our data with the largest Caucasian series with both detailed clinical and genetic information [7]. A *χ* ^2^ test was performed to compare the features between groups (classic versus atypical patients and Asians versus Caucasians). Statistical significance was defined as 2-tailed error probabilities of *P* < 0.05.

## 3. Results

### 3.1. Neuropathological Study

**Figure 1:**
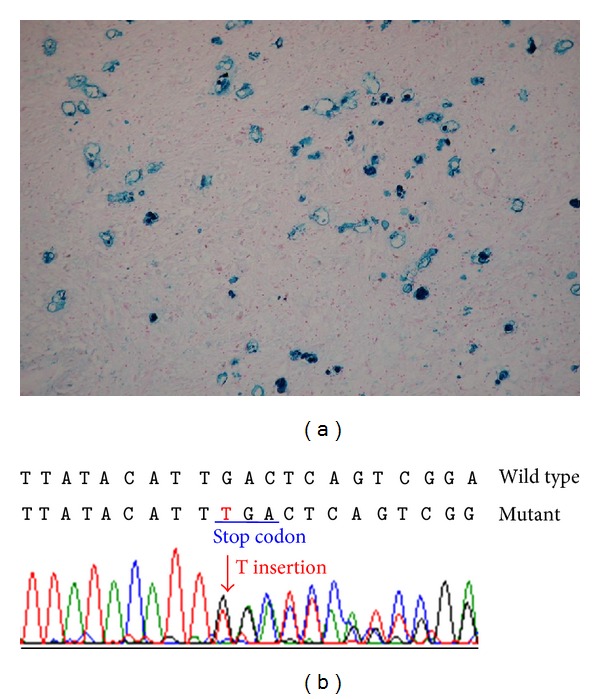
(a) The iron stain of the brain of our patient one showed diffuse granules with iron deposits in the globus pallidus. (b) Sequence chromatographs of exon 3 of *PANK2* gene showed a heterozygous T insertion (c.1408insT) leading to a stop codon and therefore a truncation of the protein at position 350 (I349X).

A postmortem autopsy was performed on patient 1. Hematoxylin and eosin stain revealed globular structures and Rosenthal fibers in bilateral globus pallidus, and iron stain showed diffuse granules with iron deposits in the globus pallidus (Figure 1(a)).

### 3.2. Genetic Analysis

DNA sequencing of the *PANK2 *gene in patient 2 revealed a heterozygous T insertion at exon 3 (c.1408insT) (Figure 1(b)), resulting in a protein truncation (p. I349X). No single nucleotide polymorphism was found among the 100 healthy unrelated volunteers (0/200 alleles). The search for copy number detection by MLPA yielded negative results. Because we failed to retrieve DNA from patient 1, the genetic proof was absent. 

### 3.3. Surface Electromyography (EMG)

**Figure 2:**
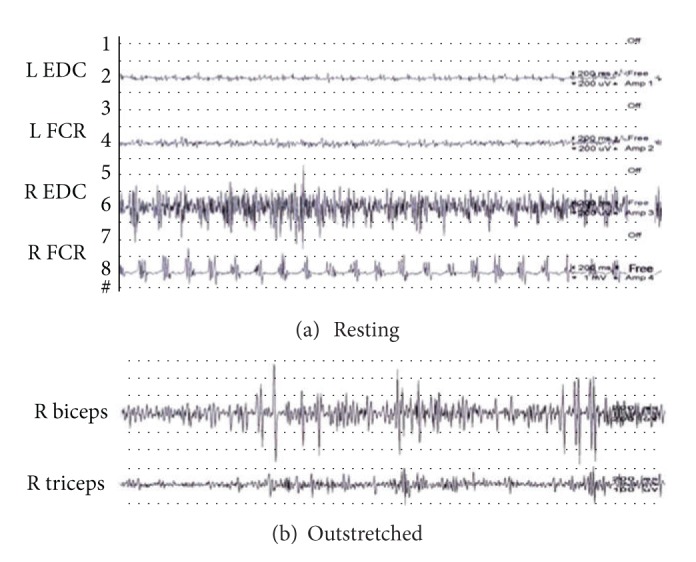
(a) Surface electromyography recording on a distal part of the arm at rest showed alternating rhythmic bursts at a frequency of 5-6 Hz. (b) Surface electromyography recording on a proximal part of the arm during outstretching showed short-duration, large amplitude arrhythmic bursts, mostly due to synchronous activities.

Figure 2 was the surface EMG recording in our patient 2. The patient exhibited 5-6 Hz tremors with alternating rhythmic bursts in the right upper limb muscles, either during forward arm posture or at rest. Synchronized bursts of the right biceps and triceps muscles were noted. The tremor amplitude was enhanced when the limbs were held outstretched because of the up-and-down excursion of the arms on abducted shoulders (wing-beating tremor).

### 3.4. Clinical Analysis

We searched the literature for patients with PKAN and included them along with our 2 patients. There were 21 patients (including 2 patients reported in this study) who fulfilled the criteria described above [8–22], including 9 with classic PKAN (1 Taiwanese, 3 Hong Kong nationals, and 5 Japanese) and 12 with atypical disease (6 Taiwanese, 1 Chinese, 4 Korean, and 3 Japanese). 

We divided these patients into classic (onset before 10 years of age) and atypical (onset after 10 years of age) groups. The clinical features and the genetic mutations in these Asian patients are summarized in Table 1. Of the 21 patients, 9 were classified as having classic PKAN (men: 7, women: 2), and 12 with atypical PKAN (men: 10, women: 2). The mean age at onset was 6.7 ± 3.0 years (range 2–10 years) in the classic group and 31.3 ± 11.8 years (range 17–51 years) in the atypical group. Ten patients (47.6%) declared that they had at least 1 family member affected with similar symptoms and that the disease was presumably hereditary with autosomal recessive inheritance. The remaining patients were presumed to be sporadic cases. 

Table 2 shows the comparison of clinical presentations of classic and atypical PKAN in Asian patients. About three-fourth of the patients had dystonia (in both the classic and atypical groups), but they showed different clinical presentations. Patients in the classic group tended to have generalized dystonia (67%, *P* < 0.05), whereas those in the atypical group more frequently had cranial (46%) or segmental dystonia (67%, *P* < 0.05). In addition, gait disturbance (89%), dysarthria (89%), and dysphagia (56%, *P* < 0.05) were the most common features in the classic group, whereas tremors (83%, *P* < 0.05) were the most prominent symptom in the atypical group. Some symptoms were more frequently noticed in patients in the classic group, such as increased deep tendon reflex (56%), mental impairment (44%), and limb chorea (44%). Parkinsonism seemed to be a more frequent feature in patients with atypical PKAN (42%) than in those with classic PKAN (11%). Some rare symptoms such as seizures, retinal degeneration, optic atrophy, and spasticity occurred sporadically but were not recorded in the majority of patients. None of the Asian patients with PKAN in either group reported significant psychiatric symptoms, autonomic dysfunction, eyelid apraxia, alopecia, myoclonus, or lower motor neuron signs, as reported in Caucasians in the literature [23, 24].

Table 3 shows the comparison of clinical presentations of Asian and Western PKAN patients. Undoubtedly, dystonia was the dominant sign in all PKAN patients, but the characteristics of dystonia differed in Asians and Caucasians. Cranial dystonia was much less common in Asians than in Caucasians (classic PKAN: 22% versus 81%; atypical PKAN: 50% versus 89%). In atypical PKAN patients, Asians tended to have segmental dystonia (67% versus 6%), whereas Caucasians were more likely to present generalized dystonia (89% versus 0%). Dysarthria and parkinsonism were found in half of the atypical PKAN patients in spite of races. In patients with classic PKAN, dysarthria was more frequently found in Asians (89% versus 50%), but parkinsonism had higher incidence in Caucasians (50% versus 11%). Finally, pyramidal signs and mental impairment were less observed in Asians in both classic and atypical groups (*P* < 0.05).

## 4. Discussion

 We herein present 2 atypical PKAN Taiwanese patients with early adulthood onset, slow progression, and typical “eye-of-the-tiger” signs by MRI. Both of them presented dominant segmental dystonia with asymmetric proximal irregular tremors over upper limbs. Patient 1 also suffered from gait disturbance, dysarthria, and parkinsonism when the disease progressed. We analyze the distinction between classic and atypical PKAN cases. Our study also summarizes the clinical and genetic data on Asian PKAN patients and demonstrates significant differences between them and Caucasians with PKAN published in the literature. 

Our study showed that subgroups of Asian PKAN patients behaved differently. Patients with classic PKAN had more generalized dystonia and bulbar symptoms, whereas those with atypical PKAN were likely to have more segmental dystonia and tremors. Gait difficulty was less common in atypical PKAN cases, possibly because the region involved by dystonia was more limited in them and mostly in upper part of the body, as in both of our 2 cases. Although not reaching statistical significance, parkinsonism was more likely to happen in patients with atypical PKAN (42% versus 12%). In general, results in previous studies were concordant with ours [3, 7] except the prevalence of tremor. Our study showed tremors were more common in atypical PKAN. In contrast, they were more prevalent in patients in the classic group according to Hayflick et al. [3]. In conclusion, patients with classic PKAN had more complex clinical features, suggesting that a higher number of systems undergo degeneration in patients in this group than in the atypical group. 

Our study also points out that Caucasians with PKAN had more complex presentations than Asians. The prevalence of pyramidal signs, mental impairment, and parkinsonism was higher in Caucasian patients. On the other hand, the features more frequently observed in Asian PKAN patients than the corresponding Caucasians were dysarthria in the classic group and segmental dystonia in the atypical group. However, the simple presentations in Asians with PKAN might in part result from limited number of cases reported and lack of comprehensive research on their clinical features.

Tremors were a prominent feature (83%) in patients with atypical PKAN. They were often asymmetric and with frequency at 5-6 Hz, as those observed in Parkinson's disease patients. However, surface EMG showed their differences. Unlike typical Parkinsonian rest tremors, they were usually more irregular and occurred with activity or were postural tremors. Besides, repeated short bursts of EMG activity tended to superimpose on the sustained activity, implicating dystonic tremors. Surface EMG documented that the irregularity and the dystonic component were key tremor features in patients with atypical PKAN.

**Figure 3:**
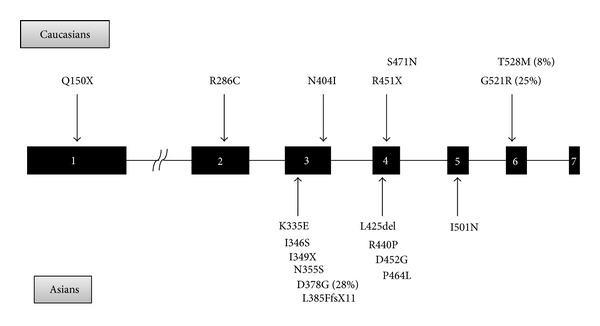
Common mutations of *PANK2* gene in Asians and Caucasians.

We identified a novel heterozygous mutation in our patient 2. He had a T insertion at exon 3 (c.1408insT), leading to a premature stop codon and hence an early truncation of the protein (p. I349X). Since pantothenate kinase 2 is the first and rate-limiting enzyme in the coenzyme A (CoA) biosynthetic pathway, this truncation may lead to a loss of function and reduction of phosphopantothenate, resulting in both CoA deficiency and accumulation of cysteine-containing molecules [4]. Most of the mutations found in patients with PKAN were missense mutations (Figure 3), but the genotypes of Asian and Caucasian patients with PKAN were very different. G521R and T528M in exon 6 were reported to account for one-third of mutations in people of European descent [3]. However, they were not found in the 12 Asian PKAN patients whose *PANK2* mutations were identified in the literature review. Instead, a mutation not found in Caucasians, D378G caused by c.1133A>G in exon 3, was the most frequent mutation (28%) among Asian patients with PKAN. Only one of these 12 Asian PKAN patients whose *PANK2* mutations were identified had one mutation outside exons 3 and 4 [22] (Figure 3). Therefore, exons 3 and 4 are hot regions for *PKAN* mutations in Eastern Asians. Most mutations gathered in the catalytic core of the *PANK2* protein including these hot spots (D378G, G521R, and T528M). Previous studies have shown that the locations of the missense mutations correlated with their biochemical and disease phenotypes. The *PANK2*-G521R mutation was mapped to the active ATP binding site and mutant proteins were catalytically inactive. *PANK2*-T528M mutation was located on the surface of the *PANK2* structure and the mutant might break the interactions between the side chains, although the enzyme activity was similar to the wild type [25, 26].

As many studies regarding rare diseases, the limitation of this study is the relatively small number of patients that may have prohibited differences in clinical features between the 2 groups from reaching statistical significance. In addition, the clinical data gathered from the literature may bring a bias due to lack of standard methods. The cases without genetic study and therefore unpublished are not included and may also lead to a bias in this study. Further prospective study with a larger number of patients and the same method is needed to confirm the results of this study.

## 5. Conclusion

Our study demonstrates the distribution of dystonia is the major distinction between subgroups of PKAN. Generalized dystonia, gait disturbance, and bulbar signs are more common in patients with classic PKAN, whereas segmental dystonia, tremors, and parkinsonism are more specific to atypical PKAN patients. Caucasian patients have more complex presentations than Asians. Exons 3 and 4 are hot regions for *PKAN* mutations in Eastern Asians. Of note, D378G is the most frequent mutation in patients from Eastern Asia, in contrast to G521R and T528M in Caucasians. Finally, surface EMG shows that the irregular, asymmetric dystonic tremors are distinguished in patients with atypical PKAN.

## Figures and Tables

**Table 1:** Clinical features and genetic mutations in Asian patients with PKAN.

Patient	Category	Onset age/sex	Cognitive decline	Bulbar signs	Gait disturbance	Dystonia	Tremor	Parkinsonism	Chorea	Pyramidal signs	Eye-of-the-tiger on MRI	*PANK2* mutation	Reference
1	Atypical	23/M	N	D	Y	S; C	Y	Y	N	N	Y	Unknown	Our patient 1
2	Atypical	17/M	N	N	N	S; C	Y	N	N	N	Y	I349X	Our patient 2
3	Atypical	46/M	Y	D	Y	S; C	Y	N	N	N	Y	D378G/D452G	Wu et al. [20]
4	Atypical	51/M	N	N	Y	S; C	Y	Y	N	N	N	D378G/D452G	Wu et al. [20]
5	Atypical	17/M	N	D	N	S; C	Y	Y	N	N	Y	D378G/I501N	Zhang et al. [22, 25]
6	Atypical	35/M	N	D	Y	C	Y	Y	N	N	Y	D378G/ R440P L385fsX11	Seo et al. [17]
7	Atypical	48/M	N	N	N	N	Y	N	N	N	Y	L385fsX11/R440P	Yoon et al. [21]
8	Atypical	21/M	N	N	N	S	N	N	N	N	Y	K335E	Chung et al. [12]
9	Atypical	29/M	N	N	Y	S	Y	N	N	Y	Y	D378G, L425del	Lyoo et al. [14]
10	Atypical	28/M	N	D; d	N	N	Y	Y	N	N	Y	I346S	Doi et al. [8]
11	Atypical	35/F	N	N	N	S	N	N	Y	N	Y	I346S	Doi et al. [8]
12	Atypical	18/F	N	N	Y	N	Y	N	N	Y	Y	N355S	Yamashita et al. [10]
13	Classic	9/F	Y	N	Y	N	N	Y	N	Y	Y	Unknown	Saito et al. [16]
14	Classic	8/M	Y	D	Y	C	N	N	Y	N	N	Unknown	Saito et al. [16]
15	Classic	4/M	N	D; d	Y	G	N	N	Y	N	Y	Unknown	Koyama and Yagishita [9]
16	Classic	9/M	Y	D	Y	N	N	N	N	Y	N	Unknown	Wakabajashi et al. [19]
17	Classic	6/M	N	D; d	N	G	N	N	N	Y	Y	Unknown	Tsukamoto et al. [18]
18	Classic	2/M	N	D; d	Y	G	Y	N	Y	Y	Y	Unknown	Ou et al. [15]
19	Classic	10/M	N	D	Y	G; C	N	N	Y	N	Y	P464L	Chan et al. [11]
20	Classic	2.5/F	N	D; d	Y	G	Y	N	N	N	Y	Unknown	Chan et al. [11]
21	Classic	10/M	Y	D; d	Y	G	N	N	N	Y	Y	Unknown	Fung and Chan [13]

PKAN: pantothenate kinsase-associated neurodegeneration; M: male; F: female; Y: yes; N: no; D: dysarthria; d: dysphagia; G: generalized; S: segmental; C: cranial.

**Table 2:** Clinical findings in Asian patients: classic versus atypical.

	Classic	Atypical	*P* value*
	*N* = 9	*N* = 12	
Age at onset (years)	6.7 (2–10)	30.7 (17–51)	<0.05
Dysarthria	89%	42%	
Gait disturbance	89%	50%	
Dystonia	78%	75%	
General dystonia	67%	0%	<0.05
Cranial dystonia	22%	50%	
Segmental dystonia	0%	67%	<0.05
Dysphagia	56%	8%	<0.05
Pyramidal signs	56%	17%	
Mental impairment	44%	8%	
Limb chorea	44%	8%	
Tremors	22%	83%	<0.05
Parkinsonism	11%	42%	
Psychiatric symptoms	0%	0%	

*Blank indicates it does not reach statistical significance (*P* ≥ 0.05).

**Table 3:** Clinical findings in patients with PKAN: Asians versus Caucasians.

	Classic patients		Atypical patients	
	Asians	Caucasians	Asians	Caucasians
	*N* = 9	*N* = 54	*N* = 12	*N* = 18
Age at onset (years)	6.7 (2–10)	4.7 (1–10)	30.7 (17–51)	18.3 (11–30)
Dysarthria	89%*	50%	42%	44%
Dystonia	78%	93%	75%	94%
General dystonia	67%	87%	0%*	89%
Cranial dystonia	22%*	81%	50%*	89%
Segmental dystonia	0%	4%	67%*	6%
Pyramidal signs	56%*	89%	17%*	83%
Mental impairment	44%*	85%	8%*	72%
Parkinsonism	11%*	50%	42%	39%
Psychiatric symptoms	0%	6%	0%	22%

*Indicates the difference between Asians and Caucasians in each group (classic or atypical) reaches scientific significance, that is, *P* < 0.05.
